# The limited usefulness of real-time 3-dimensional echocardiography in obtaining normal reference ranges for right ventricular volumes

**DOI:** 10.1186/1476-7120-7-35

**Published:** 2009-07-06

**Authors:** Erlend Aune, Morten Baekkevar, Olaf Rodevand, Jan Erik Otterstad

**Affiliations:** 1 Department of Cardiology, Vestfold Hospital Trust, Box 2168, NO-3103 Toensberg, Norway; 2 Department of Cardiology, Feiringklinikken, Feiring, Norway

## Abstract

**Background:**

To obtain normal reference ranges and intraobserver variability for right ventricular (RV) volume indexes (VI) and ejection fraction (EF) from apical recordings with real-time 3-dimensional echocardiography (RT3DE), and similarly for RV area indexes (AI) and area fraction (AF) with 2-dimensional echocardiography (2DE).

**Methods:**

166 participants; 79 males and 87 females aged between 29–79 years and considered free from clinical and subclinical cardiovascular disease. Normal ranges are defined as 95% reference values and reproducibility as coefficients of variation (CV) for repeated measurements.

**Results:**

None of the apical recordings with RT3DE and 2DE included the RV outflow tract. Upper reference values were 62 ml/m^2 ^for RV end-diastolic (ED) VI and 24 ml/m^2 ^for RV end-systolic (ES) VI. Lower normal reference value for RVEF was 41%. The respective reference ranges were 17 cm^2^/m^2 ^for RVEDAI, 11 cm^2^/m^2 ^for RVESAI and 27% for RVAF. Males had higher upper normal values for RVEDVI, RVESVI and RVEDAI, and a lower limit than females for RVEF and RVAF. Weak but significant negative correlations between age and RV dimensions were found with RT3DE, but not with 2DE. CVs for repeated measurements ranged between 10% and 14% with RT3DE and from 5% to 14% with 2DE.

**Conclusion:**

Although the normal ranges for RVVIs and RVAIs presented in this study reflect RV inflow tract dimensions only, the data presented may still be regarded as a useful tool in clinical practice, especially for RVEF and RVAF.

## Background

Right ventricular (RV) function is an important prognostic factor in both congenital and acquired heart disease [1]. In clinical practice the assessment of right ventricular dysfunction is important to a variety of conditions [2]. As early as 1982 an attempt was made to determine RV volume from the apical window by 2-dimensional echocardiography (2DE) using the Simpson's biplane method [3]. In that study, however, only "body" volumes were obtained, reflecting inflow tract dimensions which represented 55% of the total RV volume as obtained by RV angiography. In order to compensate for these problems, Levine et al. [4] incorporated a combination of apical four-chamber (for inflow) and subcostal views (for outflow). This approach, however, has not gained widespread acceptance within clinical practice. According to present guidelines, assessment of RV size is best performed in the apical 4-chamber view, with reference limits provided for both RV diastolic and systolic areas and for RV area fraction (AF) [5]. The introduction of real-time 3-dimensional echocardiography (RT3DE) has provided promising results for RV volume measurements from one single modified apical view using offline analysis with Tomtec^® ^3D software by disc summation [6].

The recent introduction of RT3DE with fast online analysis may allow bedside measurements of RV volumes and ejection fraction (EF). The purpose of the present study was to obtain normal reference values for RV volumes and EF with RT3DE parallel to reference ranges for 2DE-derived areas corrected for body surface area (BSA). In addition, a blinded and unblinded reproducibility study of the two methods was carried out.

## Methods

### Recruitment of participants

The aim was to include 15 to 20 males and females, all employed (past and present) at our hospital, per age decade from 30 to 80 years. An invitation was sent to 250 potential participants. All had to give informed written consent in order to be given an appointment for a screening visit. A total of 195 persons came to this visit, which included a 2DE examination to exclude subclinical cardiac abnormalities such as significant valvular disease (stenosis or aortic-/mitral regurgitation >1/3) or apparent abnormalities of cardiac chambers. 29 individuals were excluded for various reasons, including; antihypertensive use (n = 14), new diagnosis of hypertension (repeated blood pressure > 160/90 mmHg, n = 3), malignant disease (n = 2), a history of coronary heart disease (n = 1), treatment for arrhythmia (n = 1), congenital heart disease (n = 1), diabetes mellitus (n = 1), poor image quality (n = 1), left bundle branch block (n = 1), large atrial septal aneurysm (n = 1), chronic pericardial effusion (n = 1). Two subjects who had given their written consent did not attend the screening. BSA was calculated according to the DuBois et DuBois formula [7].

### Ethics

The study was approved by both the Regional Ethics Committee of the South-Eastern Norway Regional Health Authority and the Norwegian Social Science Data Service.

### Echocardiographic examinations

All examinations were performed using a Philips IE 33^® ^with 3D QLab advanced software (version 6) installed. According to the manufacturer this software was modified to include both left ventricular and atrial volumes as well as RV volume calculations.

2DE measurements of RV areas (A) in end-diastole (ED) and end-systole (ES) were established from the apical 4-chamber view, with simple planimetry allowing the calculation of RV area fraction (AF). Harmonic RT3DE imaging was performed using a ×3-matrix array transducer with the participant in the left lateral decubitus position. A wide-angled "full-volume" acquisition mode, in which wedge-shaped subvolumes are obtained over 4–5 consecutive cardiac cycles, was used during held respiration in end-expirium. Both an apical 4-chamber view of the RV and an orthogonal view were extracted from the pyramidal dataset in the same manner as applied for LV and atrial recordings [8]. Five anatomic landmarks were then manually initialized, including two points to identify the tricuspid valve annulus in each of the two apical views and one point to identify the apex in either view. Following manual identification of these points, the program automatically identified the 3D endocardial surface using a deformable shell model.

The orthogonal view, however, was not obtainable in all participants. In addition, the automatic border detection occasionally included various parts of the right atrium as well. The criteria for a successful recording were both a clear delineation of the RV cavity in both apical views and the exclusion of the right atrium. If that could not be obtained within three attempts then the participant was excluded from the study. Adjustments of the automatic surface detection, in order to include all trabeculae in the RV cavity that could be visualized, were done without exception in the two apical views. End-diastolic volume (EDV) was automatically computed from voxel counts. Thereafter, end-systole (ES) was selected by identifying the frame with the smallest RV dimension. Surface detection, including initialization and editing, was repeated on this frame to obtain the ESV. RVEF was calculated from these volumes.

**Figure 1:**
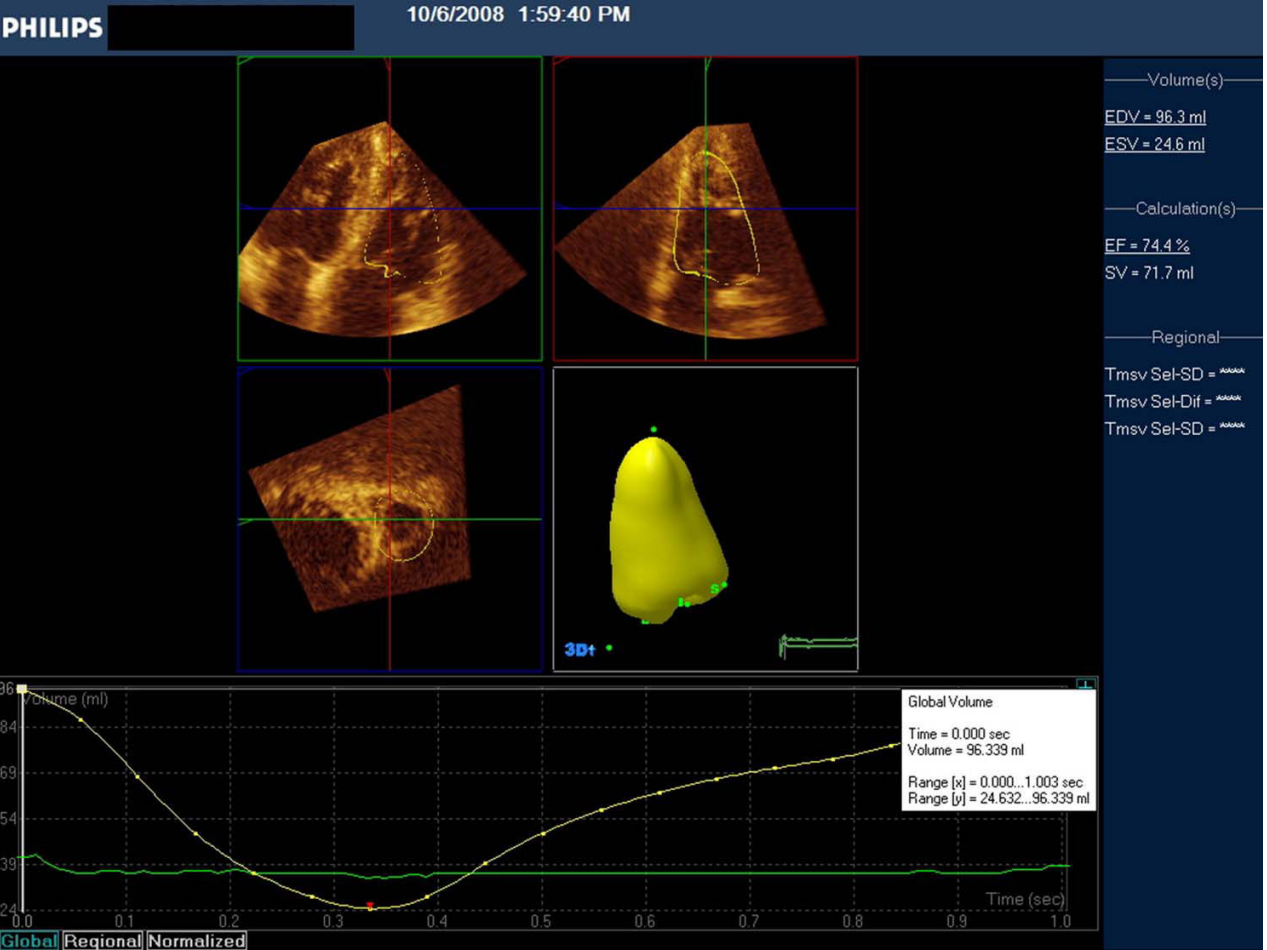
**RV tracings after editing in end-diastole**. Top: The two long axis orthogonal views. Middle the short axis view (left) and 3D model of LV (right). Bottom: time- volume curve, indicationg that these measurements are taken from the maximal dimension (white vertical line to the left).

**Figure 2:**
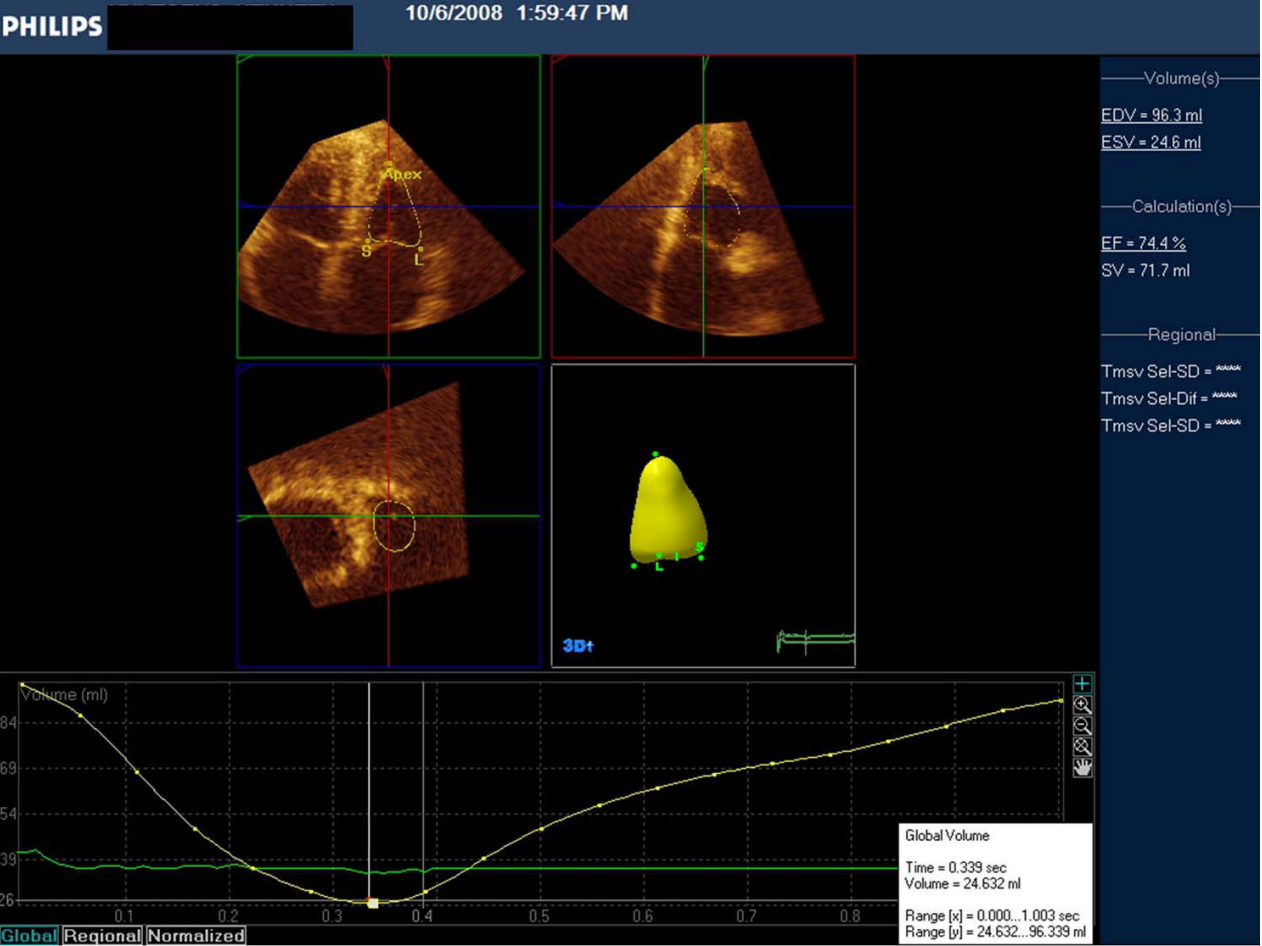
**Similar tracings in end-systole, with the time volume curve indicating the minimum dimension (white vertical line in the middle)**.

Figure 1 presents RV in ED and figure 2 RV in ES, and demonstrate the two apical views, the short axis view, the resulting 3D model of the RV cavity obtained, and the-time volume curve. The performance of these measurements took approximately five to six minutes per participant.

### Reproducibility studies

#### A: Blinded study

Two to four weeks after the initial study a repeated 2DE and RT3DE examination was performed on 20 participants (7 males and 13 females) selected at random. One examiner did all 2DE recordings (EA) and another all RT3DE recordings (JEO). Since we wanted to accurately reflect the scenario as in daily clinical practice, the repeated study was done blindly and without access to previous images or results. With both methods care was taken to include trabeculae in the RV cavity. This study only allowed us to evaluate intraobserver variability for the two investigators. In order to replicate clinical practice the presented 2DE data are taken from only one heartbeat during both examinations.

#### B. Unblinded study

This study was performed in order to optimize the reproducibility with both methods. 22 participants were picked at random (9 females and 13 males). All had given their informed consent to undergo two new separate echocardiographic examinations which were performed within a mean interval of five days. None of these persons had participated in the blinded study.

##### RT3DE

The same investigator (JEO) did all examinations and measurements. RV endocardial tracings, after manual editing from the first examination, were available during the second examination. Since only one investigator had been properly trained to carry out this method, interobserver variability data could not be obtained.

##### 2DE

These examinations and measurements were performed by the same investigator of the RT3DE recordings (JEO). Data were transferred to an EchoPAC^® ^analysis system (GE Healthcare). Offline RV tracings were performed by both investigators (JEO and EA) in order to obtain both intra- and interobserver variability. Loops and tracings from the first examination were available during the second, and all values presented are the mean of three heartbeats. To obtain the single plane measurements with three manual tracings per variable took approximately 5–10 minutes.

### Statistical analysis

Paired and unpaired t tests were used for comparison of continuous data between groups of subjects. Two-tailed p-values below 0.05 were considered statistically significant. Upper normal limit was calculated as mean +2 standard deviations (SD) and lower normal limit as mean -2SD. Pearson's correlation was used for analyses on relationship between volumes and age.

The intra- and interobserver variability for repeated RT3DE and 2DE measurements has been expressed as coefficient of variability (CV). The CV was calculated as the SD of the differences divided by the mean of the parameter under consideration [9]. All analyses were implemented using SPSS^® ^16.0 (SPSS Inc, Chicago, IL).

## Results

### Baseline characteristics and reproducibility

The characteristics of the 166 participants (87 females and 79 males) are shown in table 1. Only seven participants were aged 70 years or more. Therefore the two oldest decades are pooled. Apart from the larger body size in males, there were no statistically significant differences between males and females. A small mitral regurgitation was found in 29% of participants, whilst 6% had a small aortic regurgitation and 3% had both. Predefined successful measurements of RV volumes were obtained in 156 (95%) participants.

**Table 1:** Baseline characteristics of 166 healthy participants.

	Males (n = 79)	Females (n = 87)	P-values
Age group			
29–39 years, n	19 (24%)	20 (23%)	NA
40–49 years, n	19 (24%)	26 (30%)	NA
50–59 years, n	23 (29%)	23 (26%)	NA
60–80 years, n	18 (23%)	18 (21%)	NA
Height, m	182	168	<0.001
Weight, kg	83	69	<0.001
Body surface area, m^2^	2.05 ± 0.15	1.78 ± 0.14	<0.001
Body mass index, kg/m^2^	25.2 ± 2.8	24.4 ± 3.5	0.13
Systolic blood pressure, mmHg	127	124	0.26
Diastolic blood pressure, mmHg	79	76	0.012
Heart rate,/min	69	73	0.007
Haemoglobin, g/dL	14.9 ± 0.9	13.2 ± 0.9	<0.001
Total cholesterol, mmol/L	5.0 (4.6 -5.6)	5.0 (4.4–5.8)	0.63
High density lipoprotein, mmol/L	1.3 (1.1–1.4)	1.6 (1.4–1.8)	<0.001
Glucose, mmol/L	5.1 (4.8–5.5)	4.9 (4.6–5.2)	0.001
HbA_1_C, %	5.3 (5.0–5.6)	5.3 (5.1–5.4)	0.31
Creatinine, μmol/L	80 (73–88)	65 (60–72)	<0.001
Alanine aminotransferase, U/L	27 (21–35)	20 (16–23)	<0.001

Categorical data are presented as n (%). Continuous data are presented as mean ± standard deviation or median (25^th^–75^th ^percentile).

### Normal ranges

#### RT3DE – RV volumes and EF (table 2)

**Table 2:** Right ventricular volumes and ejection fraction obtained with real-time 3-dimensional echocardiography (RT3DE) according to age group and gender.

	Age group						
				
	29–39	40–49	50–59	60–80	Females	Males	Total
RVEDV, ml	87 ± 24	78 ± 21	70 ± 20	72 ± 23	69 ± 20	85 ± 23	77 ± 23
RVEDVI, ml/m^2^	45 ± 11	40 ± 10	37 ± 10	37 ± 11	38 ± 10	42 ± 11	40 ± 11
RVESV, ml	34 ± 12	30 ± 12	28 ± 9	29 ± 14	26 ± 10	34 ± 13	30 ± 12
RVESVI ml/m^2^	18 ± 6	15 ± 6	15 ± 5	15 ± 7	15 ± 5	17 ± 6	16 ± 6
RVEF, %	60 ± 11	62 ± 10	59 ± 11	61 ± 10	62 ± 10	60 ± 11	61 ± 10

Continuous data presented as mean ± standard deviation. RV: right ventricular, EDV: end-diastolic volume, ESV: end-systolic volume, EF: ejection fraction, I: index.

In the entire study group upper normal values were 62 ml/m^2 ^for RVEDVI and 28 ml/m^2 ^for RVESVI, whereas lower RVEF was 41%. Lower normal values were 18 ml/m^2 ^for RVEDVI and 4 ml/m^2 ^for RVESVI, whilst upper normal RV EF was 81%. Males had higher upper normal RVEDVI and RVESVI when compared to females, whilst they had a lower reference limit for RVEF.

Weak but statistically significant negative correlations were found between age and RVEDV (r = -0.28, p = 0.001) and age and RVESV (r = -0.21, p = 0.009). RVEF, however, did not correlate significantly with age.

#### 2DE – RV areas and AF (table 3)

**Table 3:** Right ventricular areas and area fraction obtained with 2-dimensional echocardiography from a single plane four-chamber view according to age group and gender.

	Age group						
				
	29–39	40–49	50–59	60–80	Females	Males	Total
RVEDA, cm^2^	25 ± 6	25 ± 5	24 ± 5	24 ± 6	22 ± 4	28 ± 5	25 ± 6
RVEDAI, cm^2^/m^2^	13 ± 2	13 ± 2	12 ± 2	13 ± 3	12 ± 2	14 ± 2	13 ± 2
RVESA, cm^2^	14 ± 4	14 ± 4	13 ± 4	13 ± 3	12 ± 2	16 ± 4	14 ± 4
RVESAI, cm^2^/m^2^	7 ± 2	7 ± 2	7 ± 2	7 ± 2	6 ± 1	8 ± 2	7 ± 2
RVAF, %	45 ± 10	44 ± 8	44 ± 8	46 ± 9	47 ± 8	43 ± 8	45 ± 9

Continuous data presented as mean ± standard deviation. RV: right ventricular area. A: area. D: diastolic. S: systolic. I: index. AF: area fraction.

Upper normal values were 17 cm^2^/m^2 ^for RVEDAI and 11 cm^2^/m^2 ^for RVESAI. The lower normal limit for RVAF was 27%. The respective lower normal values were 9 cm^2^/m^2 ^for RVEDAI and 3 cm^2^/m^2 ^for RVESAI. Upper RVAF was 63%. Males had a higher upper normal limit for RVEDAI when compared to females, but a similar reference for RVESAI and a lower reference limit for RVAF. There were no significant correlations between age and RV areas or RVAF.

#### Reproducibility study

##### RT3DE

A. Blinded study: The CVs for intraobserver variability were 37% for RVEDV, 33% for RVESV and 26% for LVEF.

B. Unblinded study: The respective CVs were 10% for RVEDV, 14% for RVESV and 13% for RVEF.

##### 2DE

A. Blinded study: The CVs for intraobserver variability were 16% for RVEDA, 17% for RVESA and 22% for RVAF.

B. Unblinded study: The CVs for intraobserver variability (average of examiner 1 and 2) were 6% for RVEDA, 13% % for RVESA and 12% for RVAF. The CVs for interobserver variability (average of examinator 1 and 2) were 6% for RVEDA, 10% for RVESA and 11% for LVEF.

## Discussion

The present study has provided gender-specific normal reference ranges for both RV volumes and EF measured with RT3DE and 2DE-derived RV areas and AF corrected for BSA. In addition, we observed a negative correlation between age and RV dimensions with RT3DE, but not with 2DE. The intraobserver variability, however, was poor with blinded measurements using both methods, indicating that follow-up measurements of individual patients should be performed after careful inspection of previous loops and tracings of the RV endocardium. This dataset has been obtained from a large population of healthy volunteers, each of whom was carefully screened for disease, including subclinical cardiac disorders.

During the progress of this study, the outflow tract, including the pulmonary artery valves, was not visualised in any of the examinations. This corresponds with the problems reported in previous 2DE studies using the apical approach [3,4].

The notion of an underestimation of RV volumes in our study is verified from reference values in studies incorporating offline analysis allowing inclusion of the RV outflow tract. In Gopal et al's. [6] study of 71 healthy individuals upper normal values for RVEDVI were 100 ml/m^2 ^in males and 92 ml/m^2 ^in females. Their respective references for RVESVI were 53 ml/m^2 ^and 51 ml/m^2^. Their lower limits for RVEF; 30% in males and 38% in females; are, however, comparable to our data. The underestimation of RV volumes in our study may therefore have been of a similar magnitude in end-diastole and end-systole, resulting in meaningful RVEF measurements.

RT3DE using off-line analyses with dedicated software (e.g. Tomtec^®^) seem to underestimate RV volumes to a less extent compared with cardiac magnetic resonance (CMR) than the methodology used in our study [10,11].

In Kjaergaard et al's. [12] study of 54 healthy volunteers upper normal RVEDVI was 88 ml/m^2 ^in males and 80 ml/m^2 ^in females. The respective limits for RVESVI were 46 ml/m^2 ^and 38 ml/m^2^. There was a tendency for smaller volumes to be associated with increasing age. The lower references for RV volumes may be related to differences in methodology, since Kjaergaard et al. used disk summation from a short axis view whilst Gopal et al. used an apical view.

CMR is regarded as the best measure of cardiac dimensions. In Gopal et al's. study [6], CMR derived max RVEDVI was 97 ml/m^2^, max RVEDVI 53 ml/m^2 ^and min RVEF 35%. These values are quite similar to their RT3DE derived references. Maceira et al. [9] studied 120 healthy volunteers comprising of 10 females and 10 males with CMR in each decade between 20 and 80 years. Their upper normal values were 100 ml/m^2 ^for RVEDVI and 41 ml/m^2 ^for RVESVI, whilst the lower limit for RVEF was 54%. BSA corrected RV volumes were larger in males and a significant negative correlation between age and RV volumes was found. RVEF did not differ between genders but was positively correlated with age.

Hudsmith et al. [13] studied 108 healthy volunteers with CMR aged between 21 and 68 years. Upper normal values were 123 ml/m^2 ^for RVEDVI and 56 ml/m^2 ^for RVESVI, whilst lower normal RVEF was 49%. BSA corrected RV volumes were larger in males than females and tended to be smaller in participants aged >35 years.

Within these studies there are considerable inter- and intra- methodological discrepancies between upper reference values for RV volumes, despite the fact that all measurements claim to have included both RV inflow and outflow tracts. Especially concerning is the large discrepancy between the lower normal limit for RVEF when derived from RT3DE as opposed to CMR. All studies, including ours, emphasize the need for gender-specific reference values corrected for BSA. The data supporting age-related corrections are not convincing. The correlations between age and RV volumes in our study were so weak that such a correction is not deemed to be necessary.

Our 2DE results also emphasise the need to use reference values for RV areas corrected for age and BSA. The normal ranges presented in the ASE/EAS guidelines are obtained from a group of 72 adult patients aged from 15 to 76 (mean 38) years with a mean BSA of 1.75 (range 1.4 – 2.1) m^2 ^[14]. For RV areas, however, only 41 participants were included. BSA corrected reference values are not presented. Max RVAEDI (mean +2SD) was 24 cm^2 ^and max RVAESI 17 cm^2^, whereas minimum RVAF (mean -2SD) was 31%. In our study the respective values were 31 cm^2^, 18 cm^2 ^and 27%. Since the measurement techniques were quite similar, the discrepancies between the upper normal diastolic areas and RV AF may be related to the differences in the study populations. These differences might have been smaller if BSA corrected values had been available as in Weyman et al's. study [14].

The blinded reproducibility study indicates unacceptable intraobserver variability with both methods. To optimize reproducibility the unblinded approach should be introduced into routine clinical practice. The intraobserver variability of 2DE derived areas was slightly better than that of RT3DE derived volumes in the unblinded study. In addition, the interobserver variability for the 2DE method was of clinical importance, emphasizing the need for repeated unblinded measurements performed by the same investigator during follow-up examinations of RV dimensions. Due to anticipated learning curve problems, we chose to use only one experienced investigator to perform the RT3DE recordings and measurements.

### Clinical implementations

With the shortcomings of the present RT3DE technique and the limitations of this study in mind, it seems apparent that single plane 2D-derived RV areas and AF is still the preferred method for assessment of systolic RV function. In adult cardiology, patients eligible for such assessment are those with congenital disorders, acute and chronic pulmonary disease, RV infarction, valvular disease and heart failure. RVAF has been shown to be an important prognostic factor in patients with left ventricular dysfunction after acute myocardial infarction [15] and in patients with severe left ventricular systolic dysfunction after coronary artery bypass grafting [16]. Whether RT3DE measurements as performed in the present study will provide additional information remains to be proven in future studies.

### Study limitations

Given that the age distribution of participants was not as wide as we had aimed for it cannot be ruled out that the age-related correlations presented might have been stronger with a wider age range. It must be stressed that the normal reference data set presented with both methods reflect the RV inflow tract, since inclusion of the RV outflow tract was not possible with the apical approach and methods applied. Due to the importance placed upon examiner experience with this new method we have not provided interobserver variability for RT3DE derived volumes. Due to imitated capacity in our CMR-laboratory, it was not possible to obtain RV volumes with that method as reference.

## Conclusion

RT3DE with automatic border detection and fast online analysis is hampered by the underestimation of RV volumes when compared with MRI and RT3DE with offline analysis. The RVEF data presented may be more realistic for RV function since the error in volume determination may be equally pronounced in ED and ES. The serial reproducibility is only acceptable if previous recordings and tracings are available. 2DE measurements of RV areas and AF are simple to perform and are fairly accurate and reproducible provided the unblinded approach is practiced. Both methods are suitable as a bedside tool for immediate clinical decisions. The present study has expanded the knowledge of normal RV dimensions and contractility obtained with apical RT3DE and 2DE recordings from a large series of individuals, allowing the presentation of BSA and gender corrected values.

## Competing interests

The authors declare that they have no competing interests.

## Authors' contributions

EA participated in the design of the study, performed echocardiographic examinations, did all statistical analyses and drafted the manuscript. MB performed echocardiographic examinations and critically revised the manuscript for important intellectual content. OR participated in interpretation of the data and critically revised the manuscript. JEO participated in the design of the study, performed echocardiographic examinations and drafted the manuscript. All authors read and approved the final manuscript.
